# Effect of Oral Midazolam Premedication on Children’s Co-operation Before General Anesthesia in Pediatric Dentistry

**Published:** 2014-09

**Authors:** Nasser Kaviani, Mina Shahtusi, Maryam Haj Norousali Tehrani, Sara Nazari

**Affiliations:** a MS. Anesthesiologist, Dept. of Oral and Maxillofacial Surgery, Torabinejad Dental Research Center, School of Dentistry, Isfahan University of Medical Sciences, Isfahan, Iran.; b Postgraduate Student of Pediatrics, School of Dentistry, Isfahan University of Medical Sciences, Isfahan, Iran.; c Dept. of Pediatric Dentistry, Torabinejad Dental Research Center, School of Dentistry, Isfahan University of Medical Sciences, Isfahan, Iran.; d Dentist, Isfahan, Iran.

**Keywords:** General anesthesia, Midazolam, Pediatric dentistry, Premedication

## Abstract

**Statement of the Problem:** Premedication is expedient in reducing the psychological trauma from recalling the unpleasant pre-anesthetic phases, hence, inducing a trouble-free anesthesia.

**Purpose:** This study aimed to determine the effectiveness of oral midazolam in co-operation of the subjects before general anesthesia and in recalling the pre-anesthetic phases, performed on children candidate for dental treatment under general anesthesia.

**Materials and Method: **In this prospective clinical trial study, 62 healthy non-cooperative children, candidate for dental treatment under general anesthesia, were randomly divided into study and control groups. The children received 20ml orange juice, 20 minutes before starting the anesthesia. The juice of the test group contained 0.5mg/kg of midazolam and that of the control group included no medication. The induction and the maintenance process of anesthesia were similar in both groups. The manner of subjects when separated from parents, their cooperation during intravenous catheterization, and recalling the pre-anesthetic events were recorded. Data were analyzed by adopting chi-square and Mann-Whitney tests.

**Results:** Most of the children in the test group had a comfortable separation from parents, restful IV catheterization and 90% of the subjects did not recall the pre-anesthetic events.

**Conclusion:** Under the circumstances of this study, it could be concluded that 0.5mg/kg oral midazolam premedication is effective for comfortable separation of children from parents and restful IV catheterization and also forgetting the pre-anesthetic events.

## Introduction

Managing a very anxious pediatric dental patient is one of the challenges of dentists. Sedation and general anesthesia are acknowledged in controlling the non-cooperative patients [1-5].

Non-cooperative children and those with physical disorders or mental retardation are the proper candidates for general anesthesia [6-7]. In the 19th century, 25% of the non-cooperative pediatric dental patients received general anesthesia to undergo dental treatments [9-10].

Currently, most of the hospital pediatric dental procedures are performed under outpatient general anesthesia. One of the drawbacks of this method is the challenging separation of children from their parents which may consequently cause a psychological trauma in the children [10-11].

The anxiety with separation is experienced enormously at age one whilst the genetic, personality, the previous experiences and the anxiety of parents are the factors concerned in the severity of the children anxiety [11]. Recalling the early phases of anesthesia which begins with the placement of anesthesia mask and follows with the smelling an unpleasant anesthetic gas is an unlikable experience [12-13].

Some medications such as antihistamines and benzodiazepines [14] are used as pre-anesthetic tranquilizers [14]. Midazolam, in the oral route, is a common pre-anesthetic medication [15]. Cox et al. [16], in the review of 30 articles, concluded that midazolam had the least side effects at the recovery time when used as the pre-anesthetic medication. They also stated that this medication could reduce the anxiety caused by separation from parents or the induction of anesthesia [16].

Some studies reported the doses of 0.25mg to 1.0mg/kg for midazolam, when employed as a pre-anesthetic tranquilizer [17-18]. The actual dose of oral midazolam premedication is determined on the basis of the duration of surgery and the severity of the child’s anxiety. McMillan et al. [19] and Feld et al. [20] reported that 0.75mg/kg of oral midazolam premedication was the most effective and the safest dose, though the age of a child is the most imperative factor in determining this dose. They concluded that the children could be separated easily from their parents 10 minutes after prescribing 0.5 to 1 mg/kg of oral midazolam whilst the maximum effectiveness appeared in 20 to 30 minutes [18-22]. The effect of oral midazolam is also reported to start in 10 and end in 45 minutes [22].

Midazolam is better tolerated when prescribed it in an apple juice [21]. Despite all advantages, oral midazolam in the dose of 1 mg/kg may cause severe respiratory depression and also delayed recovery [19-25], while none of these disadvantages were experienced when the dose of 0.75mg/kg was prescribed. Moreover, deeper levels of tranquility were observed in some cases [24]. Midazolam is used as the premedication in more than 90% of surgical operations in the United State of America [9].

Patients may remember the surgery events or procedures if the anesthesia is not induced deep enough [26]. Recalling the events of surgery is uncommon and the incidence is reported to be 0.4% to 0.8% in adults and 1.4% to 2.7% in children [27-28].

The inherent anxiety of pre-anesthesia and the recalling of the pre-anesthetic events could proceed to psychological trauma and affect the quality of children’s life, therefore, evaluating the effect of pre-anesthetic oral midazolam, on controlling these problems, seems to be indispensable.

## Materials and Method

In this triple-blinded prospective clinical trial study, 62 healthy (ASAI, Frankel **-**) children with the age range of 4 to10 years were recruited to receive dental treatments under general anesthesia. The study was performed in the surgical operating room at the School of Dentistry, Isfahan University of Medical Sciences, Isfahan, Iran. The written consent forms were taken from the parents after being completely informed about the details of the study procedure. All the children were examined by the anesthesiologist and received similar information. The children were divided into the test and the control groups, using the stratified random selection method. The children of the test group received 20 ml orange juice containing 0.5mg/kg of oral midazolam (Tehran Chemie Pharmaceutical co.; Iran). The control group received the orange juice only without any medication. Children were separated from their parents 20 minutes later. The ease of separation was recorded based on the parental separation anxiety before the dental surgery (PSAS) [29]. In this measurement, we scored 1 as easy separation; 2 when child whimpered but was easily reassured and not clinging; 3 when child cried and could not be easily reassured but not clinged to parents; and 4 when cried and clinged to parents. Although this scoring is not published in psychometric data, we employed PSAS method regarding a published study [30].

Weldon et al. [31] recommend scoring criteria, which was employed in this study: a PSAS score of 1 or 2 was classified as an acceptable separation whereas the scores of 3 or 4 were considered difficult separations from the parents.

The children under study were then laid on the operating Table. Intravenous catheterization has been performed if the child exhibited cooperation; otherwise the child was managed to inhale slowly a mixture of oxygen, N2O and Isoflurane before IV catheterization. Intravenous anesthesia induction with thiopental Na, Fentanil and Atrecurium followed by tracheal intubation was performed. The sedation was maintained with O2/N2O and Isoflurane during anesthesia, and the children were monitored in a standard approach. At the end of anesthesia, the children were transferred to the recovery room and were monitored. Subsequently, the oxygen therapy with face mask, clinical observation and pulse oximetry was performed. When they were able to earn a post-operative score of 9 or higher based on post anesthesia discharge scoring system (PADS, Table 1), the children were asked if they recall the pre-operative and operating procedures [32]. The recalled events such as hand contact during IV catheterization, hearing, seeing and the placement of mask to induce anesthesia through inhalation, were recorded on a chart which was prepared by adopting the method enrolled in other studies [27-28].

Data were then analyzed using chi-square and Mann-Whitney tests.

**Table 1:** Post Anesthetic Discharge Scoring System (PADS)

Vital Signs	2= within 20% of preoperative value 1= 20%-40% of preoperative value 0= > 40% preoperative value
Activity and mental status	2= Oriented x3 AND has a steady gait 1= Oriented x3 OR has a steady gait 0= Neither
Pain, nausea and/or vomiting	2= Minimal 1= Moderate, having required treatment 0= Severe, requiring treatment
Surgical bleeding	2= Minimal 1= Moderate 0= Severe
Intake and output	2= has had PO fluids AND voided 1= has had PO fluids OR voided 0= Neither

Total pads score is 10; Score ≥9 considered fit for discharge PO = oral administration

## Results

The mean value of age of participants the test and control groups was 5.797 and 5.797 years respectively. The values of age, durations of anesthesia and recovery was compared between test and control groups (Table 2).

**Table 2:** Age, anesthseai and recovery time in two groups

	**Group**	**Main±SD**	**Max**	**Min**
Age	Control	5.79±1.62	5.8	5.7
	Study	1.5±6	6	5.7
Anesthesia Time(Min)	Control	65.47±25.6	65	60
	Study	71±24.89	71	60
Recovery Time(Min)	Control	82.65±28.4	83	60
	Study	101.66±23.61	102	60

The Mann-Whitney test showed that the ease of separation from parents was better in the study group compared to the control group (Table 3). Induction of anesthesia in 90% of study and 15.6% of the control group was by performed by IV catheter and the remainder by inhalation method. The chi- square test showed that the difference in the induction route between two groups was statistically significant (*p*< 0.001). Recalling pre-anesthesia events was observed in 10% of the test and 81.3% of the control group. This difference was statistically significant (*p*< 0.001). The differences in recalling hand contact during IV catheterization (*p*= 0.062), hearing, and seeing between two groups were not statistically significant (*p*= 0.062) although the recalling of face contact during the placement of anesthesia mask was significantly lower in the study group (*p*< 0.001). There was no correlation between age and duration of anesthesia or recovery in two groups. Pearson’s correlation coefficient showed no relation between durations of recovery and anesthesia (r= 0.352, *p*= 0.005). Based on the result of Student t-test, there was a significant relation between the duration of recovery and the mean value of age of two groups when they were combined.

**Table 3:** Separation from parent in two groups

**Separation problems**	**Study Group**		**Control Group**		**P value**
	**Number**	**%**	**Number**	**%**	
Difficult separation	11	36.7	30	93.8	0.001
Acceptable separation	19	63.3	2	6.3	0.001

## Discussion

In this study, the ability of recalling the pre-anesthetic events were compared between the 30 children of test group who received oral midazolam premedication (0.5mg/kg) and the 32 children of the control group. The drawback of oral administration of midazolam is that it is extremely bitter. In this study we used orange juice as a carrier since we found that it easily available and convenient to use. 

 Higher percentage of intravenous induction of anesthesia in the study group indicates the higher cooperation in this group compared to the control group.

Anxiety has been associated with postoperative adverse events such as increased pain and negative behavioral changes; therefore, reducing the preoperative anxiety must be one of the most imperative concerns in pediatric anesthesia [10-11]. Hence, the use of tranquilizers and anesthetic medications in management of anxious and non-cooperative children is inevitable [21].

Compared to other benzodiazepine and non-benzodiazepine medications, midazolam is reported to be equally or more effective when used as premedication/preoperative sedation [33]. The premedication with midazolam does not prolong the discharge time from the hospital and its effectiveness and safety have been extensively studied [19, 22]. Enno et al. reported that premedication with midazolam was effective in reducing both separation and induced anxiety in children with minimum consequence on the recovery period [33].

The current study, in line with other studies [19, 22], showed that the ease of separation from parents was better in the study group who received the midazolam compared to the control.

Anterograde amnesia is defined as the absence of recalling the events occurring from the time of administration of a medication onwards [34]. Midazolam influences the memory processes by impairing the ability to acquire new information. The medication provokes its effects on initial stimulus encoding or memory storage processes rather than the retrieval process [34].

In this study, the incidence of recalling pre-anesthetic phases in the control group was 81.3%, while it was 10% in the test group. This difference was statistically significant. Recalling the placement of mask to induce anesthesia through inhalation shows the consciousness of the child. The children in control group recalled the placement of anesthesia mask. It shows that they were significantly more conscious compared to test group who received oral midazolam. The incidence of recalling hands contact during IV catheterization was not significantly different in the two groups. If the samples were more the difference might be significant. The incidence of recalling sounds and images at the beginning of anesthesia in two groups was not significantly different.  

Orally administered midazolam can be given in a dose of 0.25 to 1 mg/kg up to a total dose of 20 mg depending on the duration of surgery and the anxiety level of the child [17-18]. The results of this study which are consistent with McMillan’s [19] and Levine’s [22], show the efficacy, safety, and sufficiency of 0.5mg/kg oral midazolam premedication.

Sufficient doses of midazolam for different kinds of surgery are variable. In the present study due to limited dental treatments the amounts of stress and anxiety were minimal; therefore prescribing 0.5mg/kg oral midazolam premedication was seemed to be sufficient. Sheta et al. [35] reported that oral midazolam can be administered in a dose of 0.25 to 1 mg/kg up to a 20 mg regarding the duration of operation and the level of the child anxiety. They concluded that oral midazolam as premedication was adequate, effective and safe in 0.75 mg/kg dose, whilst the dose of 0.50 mg/kg dose was less helpful. They described that a dose of 1 mg/kg could produce more sedation over a 0.75 mg/kg dose but might delay the recovery time, therefore, compromising the safety of the child [35].

The study of Feld et al. [20] showed a better anxiolysis with administration of 0.75 mg/kg dose of orally administered midazolam when compared to 0.25mg/kg and 0.5 mg/kg doses or placebo. They concluded that the use of a 0.7 mg/kg dose of oral midazolam would not cause respiratory depression or upper airway obstruction, although in some children produced over sedation. Nonetheless, the finding of the current study is not in line with their results. The different results might be due to the dissimilarities in drug preparation, employed sedation scales, patient characteristics and the inequality in the time spent between the administration of premedication and separation from parents.

Clinical sedative effects of midazolam occur within 5 to 10 minutes of oral midazolam administration; the maximum effect is accomplished in 20 to 30 minutes. The sedative effects diminish within 45 minutes in most cases. Midazolam has been administered orally in the doses of 0.2–1 mg/kg, having 20- 30 minutes onset of action [35]. The maximum effect of midazolam in the study of Kupietzky et al. [23] appeared in 20 minutes, the finding which is in harmony with our results.

Moreover, the results of the current study are in line with the finding of and Nadin et al. [36] study in which the events were forgotten after taking midazolam. It is noteworthy that Reeves et al. [37] reported the mental function returns to normal after 4 hours of giving oral midazolam as a premedication.

Recalling the events in surgical time has been observed in 1.2 to 2.7% of cases [27] while recalling the sounds and images in surgical time of the present study was observed in 9.4% and 6.3% of control group respectively, but not in the study group.

## Conclusion

The present study showed that taking 0.5mg/kg oral midazolam 20 minutes before starting the process of anesthesia makes the separation of child from parent(s) easy. It also has positive effect on the cooperation of child with anesthesiologist, and prevents the child from recalling the pre-anesthetic events.
